# Effect of Physiotherapy Treatment in the Autonomic Activation and Pain Perception in Male Patients with Non-Specific Subacute Low Back Pain

**DOI:** 10.3390/jcm10081793

**Published:** 2021-04-20

**Authors:** Vanesa Abuín-Porras, Vicente Javier Clemente-Suárez, Gonzalo Jaén-Crespo, Emmanuel Navarro-Flores, Helios Pareja-Galeano, Carlos Romero-Morales

**Affiliations:** 1 Faculty of Sport Sciences, Universidad Europea de Madrid, Villaviciosa de Odón, 28670 Madrid, Spain; vanesa.abuin@universidadeuropea.es (V.A.-P.); gjaencrespo@gmail.com (G.J.-C.); helios.pareja@universidadeuropea.es (H.P.-G.); carlos.romero@universidadeuropea.es (C.R.-M.); 2 Grupo de Investigación en Cultura, Educación y Sociedad, Universidad de la Costa, 080002 Barranquilla, Colombia; 3 Frailty Research Organized Group (FROG), Department of Nursing, Faculty of Nursing and Podiatry, University of Valencia, 46001 Valencia, Spain; emmanuel.navarro@uv.es

**Keywords:** nervous system, low back pain, physical therapy

## Abstract

Introduction: Physiotherapy treatment is a common intervention for low back pain (LBP) patients. These interventions have been related to physiological effects in the central nervous system. Thus, the aim of this study was to analyze the effect of physiotherapy treatment in patients with LBP in the autonomic nervous system activation and subjective pain perception of patients. Methods: A total of 30 male subjects diagnosed with non-specific subacute LBP received a 50 min session consisting of (a) a manual therapy based on joint mobilization and soft tissues techniques in the lumbo-pelvic area, (b) a stretching program, and (c) motor control exercises of the core muscles. The autonomic modification of participants was assessed prior to and after the physiotherapy treatment. Results: Heart rate variability (HRV) analysis reported a significant increase in average RR (*p* = 0.001), RMSSD (*p* = 0.008), LRMSSD (*p* = 0.001), SDNN (*p* = 0.005), and PNN50 (*p* = 0.024) after the session. Frequency-domain measures showed a significant increase in LF (*p* = 0.030) and HF (*p* = 0.014), and a decrease in LF/HF ratio (*p* = 0.046). A significant decrease was found in minimum HR values (*p* = 0.001) and average HR (*p* = 0.001). Moreover, maximal HR decreased its value from 116.7 ± 26.1 to 113.7 ± 40.8 after intervention. In addition, subjective pain perception (VAS scores) was significantly lower (*p* = 0.001) in the post-session assessment. Conclusions: Physiotherapy treatment produced an increase in parasympathetic nervous system activation and a decrease in subjective pain perception in non-specific subacute LBP patients.

## 1. Introduction

Through the past decades, noninvasive treatments in the management of chronic low back pain has been a topic of research [1,2]. Amongst the most studied noninvasive methods, physiotherapy has been commonly included in LBP management protocols [3]. Physiotherapy treatment for LBP generally includes a combination of mobilization, massage, and different manual therapy techniques [4,5], combined with active therapies such as motor control exercise [6,7,8], Pilates exercise [9], and others [10]. Some authors, analyzed different approaches offered for the treatment of back pain, suggesting the use of massage as a complement to other therapies such as mobilization or exercise [11]. Several studies reported the effectiveness of this techniques to certain degree, especially in the short term [3,5,12,13]. Despite the high prevalence of back pain, few treatment methods have a critical evidence [14].

Changes in the activity of the autonomic nervous system after the application of certain physiotherapy techniques have been reported by some authors, especially invasive techniques such as percutaneous needle electrolysis and acupuncture [15,16,17,18,19], but also electrotherapy techniques such as interferential currents [20]. Mild vagal reactions have also been observed during the application of noninvasive physiotherapy techniques, such as craniosacral therapy [21] and massage [22,23,24].

The clinical interest of sympathetic (SNS) and parasympathetic nervous system (PNS) activity in this context relies on the role they play in the regulation of pain states, especially chronic pain. Tracy et al. performed a metanalysis in this topic, concluding that a decrease in parasympathetic activation is commonly found in chronic pain patients [25]. A common method for evaluation of autonomous system activity is the assessment of heart rate variability (HRV) [26]. In fact, in chronic pain patients, a decreased HRV is a common finding, probably influenced by a constant activation of the stress response and persistent SNS activation caused by pain mechanisms [27]. HRV has also been studied in several clinical conditions [28,29,30] and sport performance [31]. Thus, the interest on the assessment of HRV as a sign of autonomic nervous system disfunction has led to including this evaluation during the application of various physiotherapeutic techniques, such as massage [24], craniosacral therapy [21], acupuncture [15,27], spinal manipulative therapy [32], and percutaneous nerve stimulation [17,18,19]. These techniques, although very common in physiotherapy practice, are rarely used in low back pain treatment in an isolated way. To the authors’ knowledge, this study is the first to explore autonomic activation after a common combination of physiotherapy techniques usually applied in daily clinical practice for patients with low back pain.

The aim of this study was to analyze the effect of a physiotherapy treatment in patients with LBP in the autonomous nervous system activation and subjective pain perception of patients. The initial hypothesis was that the physiotherapy treatment would decrease the sympathetic autonomous activation and pain perception of patients.

## 2. Methods

### 2.1. Design

The present study was a prospective clinical trial developed from September to December 2020, following the Template for Intervention Description and Replication (TIDieR) guidelines [33].

### 2.2. Participants

In this study, 30 volunteer male patients (35.1 ± 9.2 years; 1.71 ± 0.1 m; 64.2 ± 11.1 kg; 21.9 ± 0.9 BMI) diagnosed with non-specific subacute LBP by a medical doctor were included. The recruitment was carried out by a medical doctor with more than 25 years of experience. The inclusion criteria were as follows: ≥18 years old, LBP (severity ≥ 3/10 in visual analogue scale), and a current episode of 2–12 weeks with the presence of back pain symptoms. Exclusion criteria were: fractures, inflammatory arthropathies, spine disturbances, metabolic diseases, any disc condition, other LBP interventions or treatments, and other physical or mental disturbances reported in the clinical exam [34].

The sample size for the present study was calculated by employing a one-sample *t*-test with Jpower tool (Jamovi software), with a power of 0.80, α error of 0.05, and an effect size of 0.5, resulting in a total sample of 30 subjects.

### 2.3. Ethics

The whole procedure was performed following the Declaration of Helsinki (revised in Brazil, 2013) and approved by the Ethics Committee of the European University of Madrid (Spain) (CIPI/18/074). Before starting the study, all participants were informed about the process to be carried out and gave their voluntary written informed consent.

### 2.4. Physiotherapy Intervention

All the patients went through 1 physiotherapy session consisting in a manual therapy intervention based on joint mobilization and soft tissues techniques in the lumbo-pelvic area [35]; stretching program; and motor control exercises of the core muscles—transversus abdominis, internal oblique, external oblique, and multifidus—following Ford et al.’s guidelines. All the individuals started the motor control exercises in unloaded positions, and the progression depended on good control and endurance with no pain during exercises [36]. The length of the sessions were 50 min.

### 2.5. Procedure

The autonomic modification of participants was assessed prior to and after the physiotherapy treatment. For this aim, participants were laying in a supine position for 5 min for HRV measurement in a stretcher placed in a room with controlled temperature, following previous research protocols [37].

The autonomic activation of the participants was analyzed by HRV measure using Polar V800 equipment (Polar, Kempele, Finland), following previously reported research protocols [38]. This evaluation allowed us to objectively determine the participants’ autonomic activation in a non-invasive, easily accessible, and affordable way. The intervals between successive heartbeats (RR intervals) were analyzed by the Kubios HRV software (version 2.2, Biosignal Analysis and Medical Imaging Group, University of Kuopio, Finland). Since previous research found different sensitivity depending on the HRV domain analysis and the characteristic of the context [39,40,41,42], we analyzed 2 HRV domains to detect modification in HRV in this context:

Time-domain (nonspectral): Based on the assessment of the intervals between normal heartbeats. During the statistical analysis, generally all the QRS (graphical combination of Q, R, and S waves) complexes, the duration between consecutive QRS complexes (NN interval), or the instantaneous heart rates during continuous electrocardiogram (ECG) recordings are determined [43]. We recorded the following time-domain indices: average RR intervals (ms); RMSSD (ms): the square root of the mean value of the sum of squared differences of all successive R-R intervals; LRMSSD (ms): logarithm of the square root of the mean value of the sum of squared differences of all successive R-R intervals: SDNN (ms): the standard deviation of the interbeat interval from which artifacts were removed (NN); and PNN50: the percentage of differences between adjacent normal R-R intervals more than 50 ms.

Frequency-domain/spectral measures (spectral): This analysis provides information on how the power is distributed as a function of frequency. This provides us with smoother spectral components that can be distinguished as independent from preselected frequency bands and easy postprocessing of the spectrum with an automatic calculation of low- and high-frequency power components and an easy identification of the central frequency of each component, as well as accurate estimation even on a small number of samples [44]. HF and LF (ms) were measured in order to analyze the peaks of parasympathetic, high-frequency component, frequency range: 0.15–0.40 Hz (HF), and sympathetic low-frequency component frequency range: 0.04–0.15 Hz (LF) values. The total power (ms) and LF/HF ratio was also measured.

Finally, the minimum, maximal, and average heart rate were also measured.

To assess the subjective pain perception, we employed a visual analogue scale (VAS) of 10 mm (from 0 no pain to 10 maximum pain) at baseline and immediately after the session [45].

### 2.6. Statistical Analyses

Data were analyzed using the Statistical Package for the Social Sciences (SPSS) version 21.0 (SPSS Inc., Chicago, IL, USA). The Kolmogorov–Smirnov test was used to test the normality and homogeneity of the sample in each variable. To analyze the modification of studied variables after the physiotherapy treatment, we conducted an independent *t*-test for related samples. The level of significance for all the comparisons was set at *p* ≤ 0.05, and the effect size of results was calculated with the Cohen’s D. In addition, Pearson correlation analysis was employed to test the relation between VAS and HRV variables.

## 3. Results

Data are presented as mean ± SD. RR abbreviation corresponds to the interval between successive “R” points (peaks), root mean square of successive differences (RMSSD) corresponds to the square root of the mean of the squares of the successive differences between adjacent beat to beat intervals, LRMSSD stands for the logarithm of the square root of the mean value of the sum of squared differences of all successive R-R intervals, SDNN stands for the standard deviation of all normal RR intervals, RMSSD corresponds to the square root of the mean of the sum of the squared differences between adjacent normal R-R intervals, PNN50 stands for the percentage of differences between adjacent normal R-R intervals more than 50 ms, LF stands for low frequency, and HF stands for high frequency.

The HRV analysis showed a significant increase in average RR (*p* = 0.001), RMSSD (*p* = 0.008), LRMSSD (*p* = 0.001), SDNN (*p* = 0.005), and PNN50 (*p* = 0.024) after the treatment. These data from time-domain parameters indicated a higher post-session parasympathetic activation. Concerning frequency-domain measures, the data showed a significant increase in LF (*p* = 0.030) and HF (*p* = 0.014), and a decrease in LF/HF ratio (*p* = 0.046) (Table 1).

On the contrary, a significant decrease in minimum HR values (*p* = 0.001) and average HR (*p* = 0.001) was observed, which indicated a preponderancy of PNS activation. Moreover, maximal HR decreased its value from 116.7 ± 26.1 to 113.7 ± 40.8 after intervention (Table 1). In addition, subjective pain perception (VAS scores) was significantly lower (*p* = 0.001) in the post-session assessment.

A negative, significant correlation was found between RMSSD, LRMSSD, SDNN, PNN50, total power, LF and HF, and VAS scores (Table 2), indicating a decrease of subjective pain perception related to an increase of parasympathetic activation. Moreover, a positive significant correlation was found between minimum HR and VAS scores.

## 4. Discussion

The aim of this study was to analyze the effect of a physiotherapy session in patients with non-specific subacute low back pain in terms of autonomous nervous system activation and subjective pain perception. The initial hypothesis was confirmed since after the physiotherapy session, the parameters of sympathetic autonomous activation and pain perception of the subjects decreased.

Analyzing the autonomic activation modifications after the intervention, we found how there was a direct impact on sympathetic–vagal balance. The increase in the time-domain parameters highlighted the increase in parasympathetic activation. Regarding the frequency domain, we found a general increase in autonomic activation with a significant higher total power, which was reflected in a significant increase of HF and LF variables [37]. Specifically, the sympathetic–vagal balance represented in the LF/HF ratio decreased significantly, highlighting the increased parasympathetic activation of participants, a fact also corroborated with the lower numbers in minimum and average HR parameters.

Concerning the sensibility of the measures, all the HRV parameters evaluated in this study showed a high sensibility to monitor autonomic modifications. This results differed partially with another previous studies, since depending on the context and population evaluated, different sensibility of HRV variables were found [38,42,46,47,48].

In this study, a statistically significant inverse correlation was found between RMSSD, LRMSSD, SDNN, PNN50, total power, LF and HF, and subjective pain perception. According to these results, parasympathetic activation seems to have an impact in decreasing pain perception mechanisms. Previous research highlighted that patients with chronic pain reported disturbances in sympathetic–parasympathetic balance, showing a decreased high frequency HRV, probably due to excessive SNS activation [25]. In particular, central sensitization phenomena had been studied in chronic low back pain patients [49,50,51,52]. There is still further research to be conducted to understand the complexity of chronic pain in this specific subgroup of population, but autonomic imbalance seems to play a determinant role in the perpetuation of symptoms in chronic pain [53]. The intervention conducted in the present research is a potentially useful tool to use in these patients.

Previous studies found how physiotherapy soft tissues techniques produce an increase in the parasympathetic activation [20,21,22]. Guan et al. [22] focused on the benefits on HF of massage applied to children in a pediatric intensive care unit. Their results are consistent with our study, showing a significant increase of HF (75%) and LF (56%) post-session. In this study, HF increased by 157% and LF by 41%. It has to be noted that Guan et al.’s study was conducted in critically ill children, and thus the effects they found may differ with ours due to their particular health status. Wälchli et al. [27], in their study on the effects of rhythmical massage, reported an increase of HRV, which is in line with the results of the present study. Buttagat et al. [24] explored the effects of a massage technique in HRV and pressure pain threshold in a sample of patients with back pain. Their results were concurrent with ours in terms of increased HRV parameters (total power and HF), indicating post-treatment increases in parasympathetic activation.

PNS activation produced by physiotherapy treatment could be clinically relevant in certain populations with high levels of SNS activation. The effects of stress through autonomic system analysis have been studied in several groups of subjects. A recent systematic review from Bustamante et al. [54] focused on the effects of stress in the military population and the consequences for their autonomic balance, enhancing the necessity for finding coping strategies [55,56,57]. Other authors have also explored autonomic balance in several sports [47,58,59,60,61,62,63,64], enhancing the importance of PNS activation in performance. These populations could potentially benefit from physiotherapy interventions according with our results.

Future research lines could explore the combination of physiotherapy treatment with other interventions that have been proved as useful for increasing PNS activation, such as physical activity programs [65], psychological approaches for stress control, or mindfulness [66].

Several limitations can be reported for the present study. For example, the sample was composed entirely by men. This should be taken into account in order to not extrapolate the results to the entire population. In this line, future research is needed that includes both genders. In addition, the evaluation of the HRV only in the short term must also be taken into consideration. Future research is needed with a mid- and long-time follow-up. Finally, another study limitation for the present work was that only one HRV measure was recorded.

### Practical Applications

The results of the present research highlighted the validity of HRV portable system to monitor modifications in the autonomic nervous system of patients. This system could be easily implemented in clinics as a method to control the evolution and efficiency of treatment in this populations.

## 5. Conclusions

A physiotherapy session including joint mobilization, soft tissue techniques, a stretching program, and motor control exercises of the core muscles produces an increase in parasympathetic nervous system activation and a decrease in subjective pain perception in non-specific subacute LBP patients.

## Figures and Tables

**Table 1:** Modification of heart rate variability and visual analogue scale variables after the physiotherapy treatment.

	Pre	Post	% Change	T	*p*	Cohen’s D
Average RR (ms)	738.2 ± 99.1	817.9 ± 108.3	10.8	−7.123	0.001	0.81
RMSSD (ms)	38.6 ± 14.4	52.5 ± 24.0	36.0	−2.837	0.008	0.97
LRMSSD (ms)	3.59 ± 0.31	3.89 ± 0.41	8.4	−3.654	0.001	0.97
SDNN (ms)	69.3 ± 16.8	80.1 ± 18.7	15.6	−3.069	0.005	0.64
PNN50 (%)	14.4 ± 15.8	22.0 ± 12.3	52.8	−2.377	0.024	0.48
Total power (ms)	2542.7 ± 1191.9	4325.7 ± 3354.1	70.1	−3.045	0.005	1.50
LF (ms)	2065.4 ± 1028.5	2916.2 ± 1899.1	41.2	−2.284	0.030	0.83
HF (ms)	473.5 ± 310.4	1217.2 ± 1687.5	157.1	−2.628	0.014	2.40
LF/HF ratio	5.54 ± 3.40	4.54 ± 3.10	−18.1	2.087	0.046	0.29
Minimum HR (bpm)	64.1 ± 5.8	58.2 ± 6.8	−4.7	5.769	0.001	0.50
Maximal HR (bpm)	116.7 ± 26.1	113.7 ± 40.8	−2.6	.256	0.725	0.11
Average HR (bpm)	82.3 ± 10.8	74.6 ± 9.4	−9.4	7.532	0.001	0.71
VAS	7.3 ± 0.8	5.5 ± 1.2	−24.7	8.537	0.001	2.25

RMSSD—the square root of the mean value of the sum of squared differences of all successive R-R intervals; LRMSSD—logarithm of the square root of the mean value of the sum of squared differences of all successive R-R intervals; SDNN—standard deviation of all normal RR intervals; PNN50—percentage of differences between adjacent normal R-R intervals more than 50 ms; LF—low frequency; HF—high frequency; ms—milliseconds; bpm—beat per minute; HR—heart rate; VAS—visual analogue scale.

**Table 2:** Correlation analysis between visual analogue scale and heart rate variability variables.

Variable	r	*p*
RR interval	−0.092	0.486
RMS SD	−0.447	0.000
LRMS SD	−0.451	0.000
SDN N	−0.256	0.048
PNN 50	−0.245	0.059
Total power	−0.402	0.001
LF	−0.232	0.012
HF	−0.378	0.003
LF/HF ratio	0.161	0.220
Minimum HR	0.262	0.043
Maximal HR	−0.165	0.207
Average HR	0.150	0.252

RMSSD—the square root of the mean value of the sum of squared differences of all successive R-R intervals; LRMSSD—logarithm of the square root of the mean value of the sum of squared differences of all successive R-R intervals; SDNN—standard deviation of all normal RR intervals; RMSSD—square root of the mean of the sum of the squared differences between adjacent normal R-R intervals; PNN50—percentage of differences between adjacent normal R-R intervals more than 50 ms; LF—low frequency; HF—high frequency; ms—milliseconds; bpm—beat per minute; HR—heart rate; VAS—visual analogue scale.

## Data Availability

Data available from the authors under request.
